# Parallel Sensor-Space Lattice Planner for Real-Time Obstacle Avoidance

**DOI:** 10.3390/s22134770

**Published:** 2022-06-24

**Authors:** Bernardo Martinez Rocamora, Guilherme A. S. Pereira

**Affiliations:** Department of Mechanical and Aerospace Engineering, Statler College of Engineering and Mineral Resources, West Virginia University, Morgantown, WV 26506, USA; bm00002@mix.wvu.edu

**Keywords:** robotics, path planning, obstacle avoidance, parallel computing

## Abstract

This paper presents a parallel motion planner for mobile robots and autonomous vehicles based on lattices created in the sensor space of planar range finders. The planner is able to compute paths in a few milliseconds, thus allowing obstacle avoidance in real time. The proposed sensor-space lattice (SSLAT) motion planner uses a lattice to tessellate the area covered by the sensor and to rapidly compute collision-free paths in the robot surroundings by optimizing a cost function. The cost function guides the vehicle to follow a vector field, which encodes the desired vehicle path. We evaluated our method in challenging cluttered static environments, such as warehouses and forests, and in the presence of moving obstacles, both in simulations and real experiments. In these experiments, we show that our algorithm performs collision checking and path planning faster than baseline methods. Since the method can have sequential or parallel implementations, we also compare the two versions of SSLAT and show that the run time for its parallel implementation, which is independent of the number and shape of the obstacles found in the environment, provides a speedup greater than 25.

## 1. Introduction

As autonomous vehicles and other mobile robots are integrated into our world, they need to be able to navigate in dynamic, tight, and cluttered scenarios. Example applications where both ground and aerial robots are exposed to such environments include logistics [1,2] and forest monitoring [3,4]. This indicates that the robots need to use their sensors to perceive the environment and its changes in real time [5] and then react with powerful and efficient motion plans. A major challenge for these robots is planning up to the bound of the control feedback loop frequency (e.g., ≈100 Hz) [6]. A current limiting factor for this high performance is that motion-planning algorithms spend a significant amount of time on collision checking, especially in environments with multiple obstacles. Approximately 90% of the total planning time on probabilistic roadmap (PRM) algorithms, for example, is spent on collision checking [7]. One of the main reasons for that is that these algorithms, which could be massively parallelized, execute each of their steps in sequence [7,8]. Therefore, the aim of our research is to develop efficient parallel motion planners that allow robots to move at high speeds in cluttered and dynamic environments.

**Figure 1:**
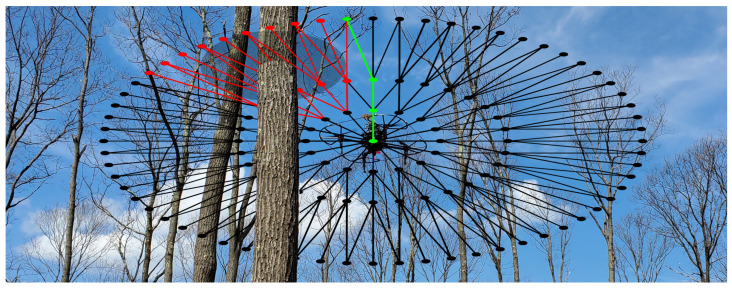
Illustration of the proposed methodology. A lattice in the sensor space encodes, in a search tree, the set of paths that the robot (i.e., drone) can take locally. The optimal path (green edges) is computed in real time by pruning the edges of the tree that are in collision (red edges) and minimizing a path cost functional on the remaining tree.

The problem considered in this paper is defined as follows: *given a planar robot equipped with a planar range sensor (e.g., LIDAR) and working in an environment populated with unknown static and moving obstacles, plan the local motion of the robot such that it can safely and quickly move among the obstacles without compromising the execution of its global task.* To solve this problem, the main challenge addressed by this paper is the real-time computation of motion plans. By solving this challenge, we will allow the robot to traverse the environment at speeds only limited by its hardware and low-level path-following controller. Thus, this paper presents the sensor-space lattice (SSLAT) motion planner, which is an embarrassingly parallel motion planner that computes paths in the sensor space of a robot in real time using unprocessed sensor data. An initial and non-parallel version of this planner was previously presented in a conference paper [9]. SSLAT is a hybrid planner that consists of two hierarchical layers. The top layer consists of a vector field, which is used to encode the global task of the robot (e.g., follow a road, traverse a forest, and track a boundary). The vector field ignores small and dynamic obstacles. In the lower layer, a lattice in the sensor space encodes a search tree that represents a set of precomputed paths. An illustration of such a lattice is shown in Figure 1. To allow for obstacle avoidance, the lattice is quickly pruned using sensor measurements. Additionally, a cost functional that indicates how aligned a path is from the vector field is used to select the best precomputed path. Therefore, SSLAT can be seen as part of a global navigation solution, provided that a global vector field is available, or as a local method for obstacle avoidance, when simple local fields are used. In the experiments presented in this paper, we mostly evaluate the ability of SSLAT to perform obstacle avoidance, but also illustrate, through a real-world experiment that it can be used to control a drone for persistent monitoring of a forest using a global vector field. The definition of global vector fields is not the focus of this paper, but it was considered in several recent publications [10,11,12,13]. SSLAT was designed to work with planar light detection and ranging (LIDAR) sensors, which makes it useful for several robots, including self-driving cars and drones. In this paper, our implementation focused on parallelizing the collision detection sub-routine (tree pruning) of the method using a NVidia^®^ CUDA^®^-enabled graphics processing unit (GPU). Tree pruning has been shown to be the most expensive component when computing plans with SSLAT. Due to the structure of the method, other sub-routines of SSLAT can be also parallelized in future implementations.

The main contributions of this paper are as follows: (1) a lattice-based method that optimizes a local, vector-field-dependent functional for obstacle avoidance and field tracking; (2) a method for generating a lattice in the sensor space of the robot that simultaneously tessellates the space and stores in a tree graph the possible paths that a robot can take locally; (3) an embarrassingly parallel strategy based on a precomputed mapping between sensor measurements and lattice edges for collision detection in parallel architectures, including CUDA. The main advantage of using a lattice to find paths that avoid obstacles and follow a vector field (Contribution 1), when compared to previous graph-based methods, is the high efficiency of obstacle detection in the lattice. While traditional graph-based approaches require several collision checks for each new node being inserted on the graph, the fixed and regular structure of the lattice allows for a constant and small number of tests. To simplify and speed up, even more, these tests, our lattice design (Contribution 2) enables a direct correspondence between the edges of the lattice and geometric shapes (triangles) in a way that a single collision detection invalidates a pair of edges and nodes of the graph. Although preliminary versions of Contributions 1 and 2 were previously presented in our conference paper [9], Contribution 3, which allows the planner to perform several collision checks in roughly the same time as a single check in the sequential version of SSLAT, has never been published before. In addition to that, the paper presents completely new simulations and experiments that evaluate the method and show its potential for real-world applications.

The rest of this paper is structured as follows: related work is presented in Section 2. The motion planner methodology is explained in Section 3. The parallelization of the algorithm is detailed in Section 4. Section 5 presents a series of simulations and real robot experiments that both illustrate and evaluate the proposed approach. The main results of this section are discussed in Section 6. Finally, Section 7 presents the conclusions and proposals for future work.

## 2. Related Work

This work is based on four main pillars, namely, motion planning using vector fields, obstacle avoidance, discretization of motion, and parallel motion planning. Vector fields represent a simple way of providing the robot with a preferred direction of motion for each point in its workspace. They can be easily computed with the use of artificial potential functions (APF) [14] or navigation functions [15]. However, using these techniques, which consider obstacles, the vector field would change every time the map of the environment changes (e.g., people or other vehicles enter the environment). An alternative strategy is then to construct the vector field ignoring the obstacles, and solve obstacle avoidance in a lower-level planner, as suggested in [16,17,18]. Thus, the vector field is used to encode the high-level specification of a task, which may be, for example, the periodic survey of a given curve [17]. Vector fields for curve circulation were proposed in [10,11,19,20].

When the obstacles are ignored by the field, local motion planning is needed to avoid obstacles and follow the vector field. The idea of modifying preexisted plans with local information is not new and was used by the authors of elastic bands [21], which deform in real time the path computed offline by a global planner. Two legacy obstacle avoidance approaches that use planar sensors to measure the surroundings of the robot are the virtual force field (VFF) [22] and vector field histogram (VFH) [23]. The former builds an occupancy grid centered on the vehicle. Repulsive forces are calculated for each cell, using the same idea of APF but corrected with a weighting function. VFH is an improved version of VFF that maps obstacles to a polar graph that has peaks in directions where the obstacles are likely to be, and valleys in directions that are probably free. In a second step, it uses heuristics to choose which valley to follow. New variations of VFH were recently proposed [24], including some that explicitly include moving obstacles [25]. Similar to these methods, our work uses sensor information directly to build knowledge about free and occupied space in the surroundings of the robot. An alternative local obstacle avoidance method is the dynamic window approach (DWA) [26], which constructs a window of allowed velocities based on data from the sensors. Although considered a legacy approach, DWA is still a very popular method. Recent works using DWA include [27], which proposes a new method for dynamic obstacles avoidance that looks for free gaps in between the obstacles to compute a trajectory and applies the DWA to safely follow such a trajectory assuming that the obstacles can move. More related to our work, assuming that a global vector field gives the desired velocity of the vehicle, the dynamic window was used to detect if the field can be followed safely [28]. If the velocity vector given by the field leads to a collision, this vector is modified before being sent to the vehicle.

Another important aspect of our approach resembles the methodologies introduced in [29,30]. In [29], precomputed relations between sensor space and planning that were embedded in collision detection circuits (CDCs) are used to prune collision nodes in a probabilistic roadmap. In [30], precomputed alternative paths generated offline are pruned using real-time data from the robot’s onboard sensor. Similarly, our method uses precomputed relations to eliminate paths in collision with obstacles. However, we chose to build a regular discretization of the sensor’s field of view using trees (graphs without cycles) that not only eliminates paths that are in collision after pruning, but also speeds up searching for the optimal path. To create the tree on the sensor space, we decided to use a lattice inspired by Bethe lattices and Cayley trees [31]. Our approach is related to ego-graphs for path planning, proposed in [32], state lattices that precompute a set of actions while accounting for dynamic constraints [33], and motion primitives [34] methods. The main difference of our proposed discretization is that the spatial representation of our lattice is carefully designed to obtain the mappings between sensor measurements and the traversable paths, thus allowing for fast pruning. Further, we use our lattice to track the global behavior (vector field) by minimizing a functional that measures how much the paths are aligned with the field, as was done in [16] using the rapidly exploring random tree star (RRT*) algorithm [35].

The use of precomputed trees using latices, besides facilitating obstacle detection, also makes the planner embarrassingly parallel, i.e., it can be easily divided into a finite number of parallel tasks [36]. There is only limited literature about the parallel implementation of motion planners. Some works with parallel architectures were conducted for sampling-based algorithms, such as rapidly exploring random tree (RRT) [37], PRM [38], and RRT* [7] and their variations [39,40]. Parallel grid-based path planners, such as the A*, were also investigated [41]. High-dimensional planners, such as R* search, were parallelized and shown to preserve all theoretical properties, including probabilistic bounds on sub-optimality [38]. The group marching tree (GMT*) was parallelized, allowing planning in real time with solutions for complex planning problems under differential constraints being found in the order of ≈30 ms on an embedded GPU [6]. A common way of parallelizing planning algorithms is to make some of their functions, such as neighbor searching [42,43] and collision checking [7,44], parallel. The authors of [44], for example, proposed a parallel approach for collision detection that was able to perform half a million collision checks per second using benchmarks problems, which is 10 times faster than prior parallel techniques. They were also able to compute collision-free paths for a great range of models (single body or multibodies) in less than 100ms for many benchmarks, which is almost 50–100 times faster than current sequential PRM planners. In the motion planning proposed in this paper, we perform parallel collision checking and obtain speed ups in the order of 80 times when compared to the sequential implementation of our collision-check approach. Moreover, we do not see any degradation in the planning efficiency when the number of obstacles and the number of beams in the LIDAR sensor increase. The next section will present the details of our approach.

## 3. Sensor-Space Lattice Motion Planner

The sensor space lattice motion planner (SSLAT) is a technique that relies on (1) a pre-computed tree graph that embeds a set of paths that a robot could take in its current field of view, and (2) sensor-to-graph mappings that can be used to quickly prune the edges of the graph that would lead to collisions between robot and obstacle. Given this, the planner is divided into two steps: an offline step that computes the graph, the sensor-to-graph mappings, and the global task (represented by a vector field), and an online step that prunes the tree and finds the free path that closely follows the vector field. These two steps are detailed next.

### 3.1. Offline Step

The first step of SSLAT, executed when the robot is initialized, is subdivided into three, as described in the following subsections:

#### 3.1.1. Computing the Lattice

**Figure 2:**
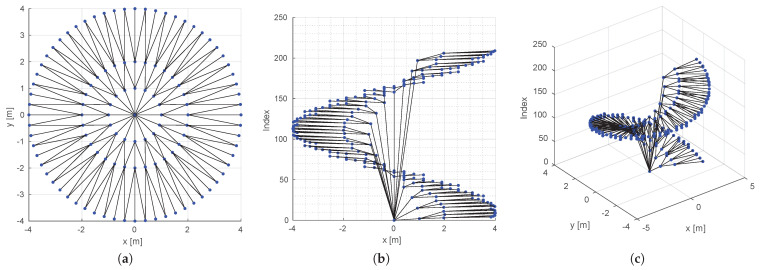
(**a**) A lattice with $\left(K,N_{T},N_{B},N_{L},r_{0}\right)=\left(2,16,3,3,1\right)$. In black, we see the edges that belong to the graph. In (**b**,**c**) it is possible to view an exploded view of the lattice, which is plotted in 3D to emphasize the fact that two non-neighbor nodes may have the same position in the plane. The graph is composed of a single tree and there is only one path from each vertex to the root.

**Figure 3:**
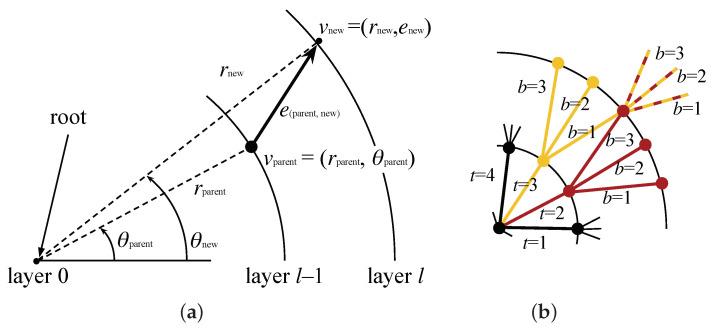
(**a**) Construction of the lattice adds vertices in layers around the sensor origin. Each new vertex ($v_{\mathrm{new}}$) is added according to its parent vertex ($v_{\mathrm{parent}}$). This process is executed recursively starting from the root node, where trunks $t=1,2,\cdots ,N_{T}$ are added connecting it to the first layer of the lattice. (**b**) For each new layer, the leaf nodes are subdivided in branches $b=1,2,\cdots ,N_{B}$. Notice that, during this process, sub-trees are created with coincident spatial representation for some of their vertices, as exemplified in red and yellow.

To encode robot local paths, we create a lattice represented by graph G(V,E), which is a planar directed tree spatially contained in the sensor space, $S\subset R^{2}$. Figure 2, at the left, shows an example of a lattice created with our methodology. The tree is constructed recursively from the root node, which is coincident with the origin of the sensor space. New vertices are created in circular layers around the root. In the first layer, the vertices are equally spaced in a circle of radius $r_{0}$ and are connected by edges coming from the root node. The number of vertices in this layer, $N_{T}$, defines the number of trunks of the tree, which is equivalent to the number of sub-trees that will be connected to the root. Each vertex of the first and subsequent layers branch out to $N_{B}$ children vertices in the next layers. The process of creation of vertices is repeated until the number of layers, $N_{L}$, is reached. The relationship of the layers’ radii is defined by the growth ratio, *K*. With these parameters (namely, $r_{0}$, $N_{T}$, $N_{B}$, $N_{L}$, and *K*), the polar coordinates (r,θ)∈S of each vertex v∈V of the graph can be found from the polar coordinates of its parent vertex as

$$\begin{array}{cc} r_{\mathrm{new}} & =K^{\left(l-1\right)}r_{0},\\ \theta_{\mathrm{new}} & =\left\{\begin{array}{c} \left(\frac{2\pi }{N_{T}}\right)\left(t-1\right)\;\mathrm{if}\;\mathrm{the}\;\mathrm{parent}\;\mathrm{is}\;\mathrm{the}\;\mathrm{root}\;\mathrm{node}\\ \theta_{\mathrm{parent}}+\left(\frac{2\pi }{N_{T}}\right)\left(\frac{b-(N_{B}+1)/2}{{\left(N_{B}-1\right)}^{\left(l-1\right)}}\right)\;\mathrm{otherwise}, \end{array}\right. \end{array}$$

where $\theta_{parent}=\tan^{-1}\left(y_{\mathrm{parent}}/x_{\mathrm{parent}}\right)$, *l* is the index of the *l*-th layer, *t* is the index of the *t*-th trunk, and *b* is the index of the *b*-th branch of each trunk (see Figure 3). Notice that there will be more than one vertex in the same (r,θ) position, but, since they come from different trunks, they are not connected. This can be observed by the yellow and red sub-trees in Figure 3 and is better visualized when the lattice is plotted in 3D with the node index as the third dimension, as shown in right plots of Figure 2. Although this generates extra data in the graph representation, keeping the nodes disconnected also keeps the graph acyclic, as a tree, which is important for the online optimization we perform using the graph.

#### 3.1.2. Obtaining Sensor-to-Graph Mappings

**Figure 4:**
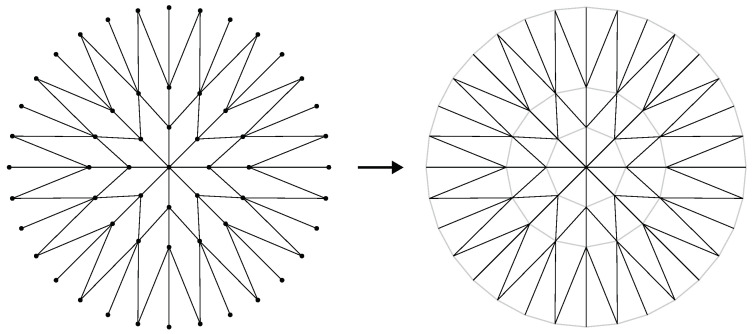
Lattice triangulation. The geometric representation of the graph is triangulated (using Delaunay triangulation, for example) to tessellate the sensor space and facilitate collision detection. The edges of the lattice are shown in black and the edges completing the triangles are shown in gray.

After generating the lattice, it is possible to create a tessellation of the region around the origin as a mesh of non-overlapping triangles that have exactly two of their sides coincident with the edges of the graph. An example of such triangulation is shown in Figure 4. Let T be the set of triangles obtained by the triangulation. By comparing the coordinates of the vertices that compose each edge and the coordinates of the triangles, it is possible to compute a map $R_{T}^{E}:T\to E$ for which each edge e∈E will have two triangles t∈T assigned to it. As the sub-trees of G may overlap spatially, this will give a one-to-many relation, where each triangle will be linked to many edges.

Now, let L be the set of range measurements coming from the sensor. For planar LIDARs, it is well known that these measurements have a particular angular distribution. For example, the sensor can have a beam at each 0.5${}^{\circ }$ around the 360${}^{\circ }$ circumference. It is then possible to compute a map $R_{L}^{T}:L\to T$ by checking which triangles can be intersected by each range measurement. Thus, each sensor range will have a list of triangles that need to be tested for collisions.

By composing $R_{L}^{T}$ and $R_{T}^{E}$ we can quickly prune the edges of the tree that are related to the obstacles detected by the sensor. Notice that, for each range measurement, only a few well-defined edges can be pruned.

#### 3.1.3. Defining a Global Task

The lattice-shaped tree presented in Section 3.1.1 is limited to be inside the field of view of the sensor. Therefore, it cannot account for the entire robot task. In the proposed methodology, we frame the global plan as an artificial vector field that is designed to follow curves in the workspace. The computation of such artificial vector fields was extensively explored in [19,45], where it was proposed that a vector field in a two-dimensional environment can be obtained by the superposition of a normal component, N, which makes the robot converge to the curve to be followed, and a tangential component, T, which drives the robot along the curve. The vector field is then defined for the coordinates ${\left(x,y\right)}_{W}$ in the world frame W as:

(1) $$v\left({\left(x,y\right)}_{W}\right)=N\left({\left(x,y\right)}_{W}\right)+T\left({\left(x,y\right)}_{W}\right).$$

**Figure 5:**
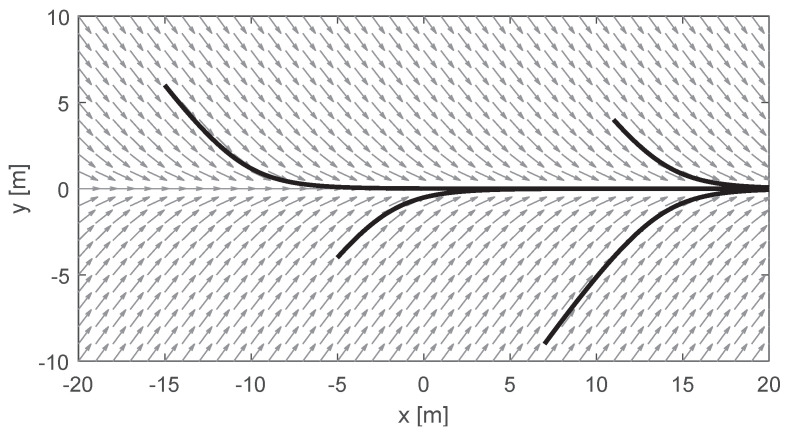
Example of a vector field that guides a robot to follow the *x*-axis. The gray arrows represent the velocity that would be given as input to the vehicle. The black curves are examples of paths that the vehicle would take by following the field.

With the correct choice of functions, it is possible to obtain a normalized vector field for a given curve. For example, Figure 5 presents a vector field that can be used to follow a straight line aligned with the *x*-axis of the world. As proposed in [19], this field can be obtained by making $N\left({\left(x,y\right)}_{W}\right)=1/\sqrt{1+f{\left(y_{W}\right)}^{2}}$ and $T\left({\left(x,y\right)}_{W}\right)=f\left(y_{W}\right)/\sqrt{1+f{\left(y_{W}\right)}^{2}}$ where $f(y_{W})=-$$\arctan c\;y_{W}$, and *c* is a convergence parameter. This parameter was set to 0.5 in Figure 5.

### 3.2. Online Step

**Figure 6:**
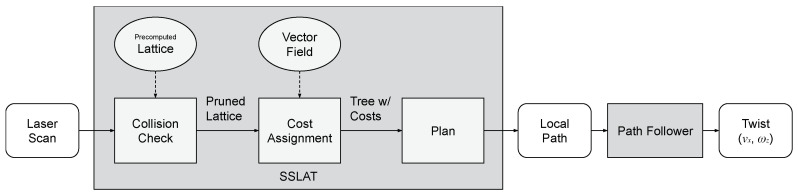
Flowchart of the method. The sequence of functions (bottom row of the chart) is run every time a new measurement (laser scan) is available.

By avoiding the necessity of generating the paths themselves, the motion planning problem to be executed online is reduced to perceiving the obstacles, pruning the tree accordingly, assigning costs to the different paths embedded in the tree, and choosing the best path to be followed. These steps are summarized in Figure 6 and explained in detail in the next subsections.

#### 3.2.1. Tree Pruning

**Figure 7:**
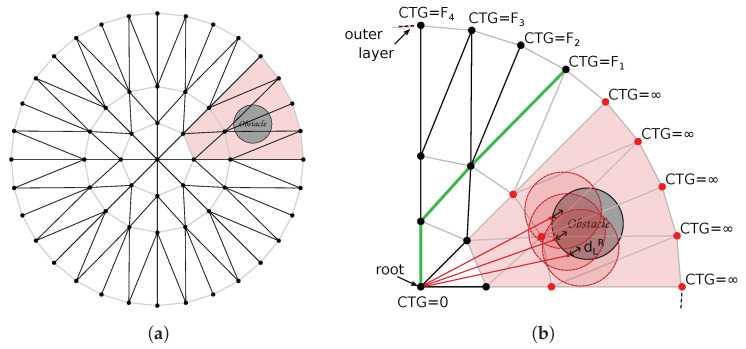
Collision detection approach. (**a**) The lattice is triangulated to facilitate collision detection. The edges of the lattice are shown in black and the edges completing the triangles are shown in gray. (**b**) Detailed view of (**a**). In red, the range sensor (e.g., LIDAR) measurements are transformed into circles in the sensor space that account for the robot dimensions. The triangles painted in light red overlap with these circles and have their edges pruned from the graph. The motion planning algorithm chooses the path, shown in green, from the remaining edges that minimizes the cost-to-go (CTG) from root to the outer layer.

Let $l_{m}={\left(x,y\right)}_{S}\in L$ be the *m*-th range measurement coming from the LIDAR sensor and $C_{m}$ be a circular region with robot radius $r_{R}$ centered at $c_{R}=l_{m}+d_{L}^{R}$, with $d_{L}^{R}$ being an offset representing the translation from the sensor frame to the robot frame. We assume, without loss of generality, since the lattice can be constructed accordingly, that both frames have the same orientation. The laser measurements are filtered, and to be considered valid, a range measurement needs to be less than the lattice outer layer radius plus the robot radius. For each triangle t∈T obtained from $R_{L}^{T}\left(l_{m}\right)$, a collision between the *m*-th valid LIDAR beam and the triangle *t* is detected if $t\cap C_{m}\neq \emptyset$. Computationally, this check can be done by a function that returns true if $\left(c_{R}\cap t\neq \emptyset \right)\wedge \left(e_{t,i}\cap C_{m}\neq \emptyset \right)$ for i=1,2,3 and false otherwise, where $e_{t,i}$ are edges of the triangle *t*. An illustration of this approach is shown in Figure 7. When a collision is detected, all the edges and corresponding vertices defined by the triangle are marked as untraversable, thus pruning the tree. The remaining tree only contains edges that can be safely followed by the robot.

#### 3.2.2. Assigning Costs

After the tree is pruned, a breadth-first search algorithm is used to assign the cost-to-go (CTG) for each vertex, assuming that the CTG of the root node is zero. The $CTG_{j}$ of the vertex $v_{j}$ is obtained by

(2) $$CTG_{j}=CTG_{i}+F\left[\xi_{i}^{j},v\right],$$

where $CTG_{i}$ is the CTG of the parent vertex $v_{i}$. The cost $F[\xi_{i}^{j},v]$ measures the codirectionality of the vector field v and the edge $\xi_{i}^{j}$. For this, we use the upstream criterion, proposed in [16,46] as the following functional:

(3) $$F\left[\xi_{i}^{j},v\right]=\int_{0}^{1}\left(1-\frac{\xi_{i}^{{}^{\prime }j}\left(\tau \right)}{∥\xi_{i}^{{}^{\prime }j}\left(\tau \right)∥}\;\cdot \;\frac{v(\xi_{i}^{j}\left(\tau \right))}{∥v\left(\xi_{i}^{j}\left(\tau \right)\right)∥}\right)∥\xi_{i}^{{}^{\prime }j}\left(\tau \right)∥d\tau ,$$

where $\xi_{i}^{{}^{\prime }j}\left(\tau \right)=\partial \xi_{i}^{j}/\partial \tau$ is the first derivative of the edge with respect to the spatial parameterization variable τ. Notice that, in our case, the derivative of each the edge is constant. Using this functional, the cost for the case in which the path is parallel to the field is zero, and the cost for the anti-parallel case is equal to twice the length of the path. Hence, the CTG of each node in the tree will be as low as the path from the root to that node is “close” to the vector field.

#### 3.2.3. Searching for the Optimal Path

After costs are assigned for each reachable vertex of the tree (some vertices are not reachable after the tree is pruned), the vertices that belong to the outer layer are sorted by their CTG. By selecting the node with minimum cost and following the back-pointers of the tree to the root node, we extract the best path of the tree. If all the vertices in the outer layer are not reachable, the precedent layer is searched for the minimum value. The process is repeated if no vertices are reachable in this layer. A minimum cost can always be found because this procedure can be repeated until the root vertex (current position of the robot), which has zero CTG. In this case, the robot will stop and return failure.

## 4. Parallelization

Due to its structure, the parallelization of the SSLAT algorithm is very natural. There are two bottlenecks in its serial implementation: tree pruning and cost assignment. In this paper, we focus on the parallelization of the pruning function, more specifically, in its implementation using the parallel computing platform CUDA [47] for computation using a graphics processing unit (GPU). By using CUDA, it is possible to define kernels, an expanded version of C++ functions that can be called in parallel by a determined number of threads. Threads are grouped into blocks, and a group of blocks forms a grid [48]. In the algorithm proposed in the last section, each valid LIDAR beam is tested for collision with each triangle in the lattice. Each of the laser measurements can be tested independently. So, to make the algorithm parallel, we create a kernel that takes as inputs the coordinates of the range measurements, the coordinates of the triangles, and the pointer to a list of length equal to the number of triangles that contains binary data representing the collision state. The list is kept in global memory, and it is initialized with 0 (no collision). The kernel runs in parallel, and each thread will either do nothing in the case that no collision is detected or change the data of the respective triangle in the list to 1 if it detects a collision.

**Figure 8:**
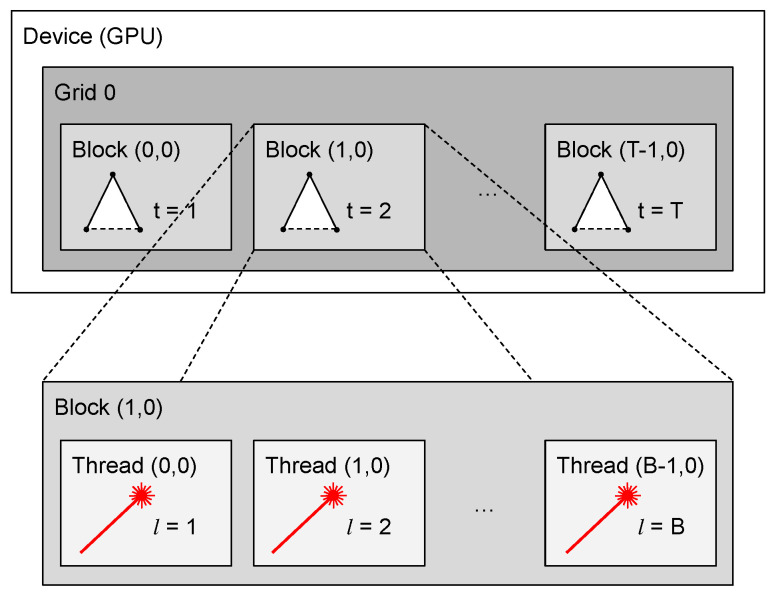
Parallel collision check approach. Triangles are organized in blocks and laser scans are organized in threads of these blocks. All collision checks are run simultaneously.

The number of blocks per grid and threads per block that will be executed in parallel need to be passed to the kernel. On the GPUs that are available at this time, a thread block is limited to a maximum of 1024 threads. The number of triangles is not limited, since it varies according to the parameters chosen to generate the lattice. The number of laser beams is tied to the sensor hardware (e.g., it is usually limited to less than 1024 for conventional planar LIDARs). Hence, in this work, we choose each block to execute *B* threads, where *B* corresponds to the number of valid laser beams, and execute tests on triangles on a grid with *T* blocks, where *T* is the number of triangles. This architecture can be seen in Figure 8. Both grid and blocks are chosen to be uni-dimensional.

The kernel operates three checks: (1) if the vertices of a given triangle are within the circle designated by the range measurement, (2) if the circle center is within the triangle, and (3) if the circle intersects any of the edges of the triangle, as described in Section 3.2.2. If any of these checks is true, it changes the status of the triangle to 1, denoting the existence of collision.

Disregarding the memory copy time of the LIDAR scans from the host (CPU) to the device (GPU) and the output back to the host, all collision checks are expected to be performed in roughly the same time as one single collision check. Nevertheless, the memory copy can be considered a great part of the time spent in the parallel implementation. Our implementation, evaluated in the next section, uses allocate unified memory, which is accessible from CPU or GPU. This further simplifies the parallel implementation.

## 5. Experiments

This section presents simulations and real robot experiments that illustrate and evaluate the proposed strategy. The experiments shown in this section complement the ones presented in our conference paper [9], which include simulations in very dense forests and a real-world demonstration with an iRobot Create robot localizing itself using simultaneous localization and mapping (SLAM). Videos of these experiments can be seen at https://youtu.be/Axn7XRimgFU (accessed on 21 June 2022). Our new experiments are presented next.

### 5.1. Simulations with a Ground Robot in Static Environments

This subsection shows the potential of our methodology to guide a ground robot in a environment with obstacles. We used part of the Benchmark for Autonomous Robot Navigation (BARN) dataset [49] to compare the performance of the method with other strategies. Although this dataset is meant to be used by complete navigation stacks that include mapping and global motion planning, we found that SSLAT is able to find solutions for most environments tested.

#### 5.1.1. Experimental Setup

**Figure 9:**
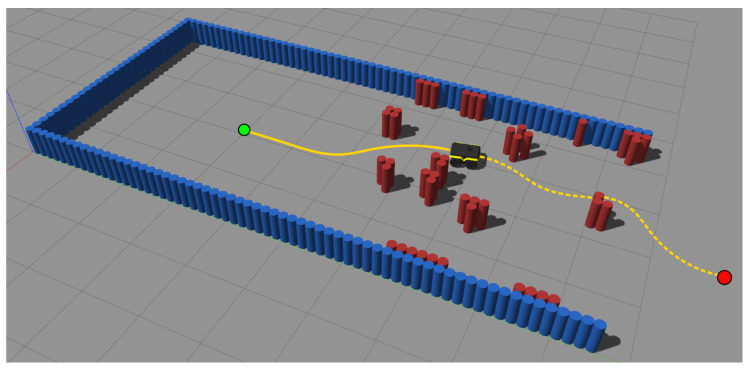
Environment #6 of the BARN dataset [49]. The objective is to exit the enclosed environment (blue cylinders), going from the start position (green dot) to the goal region (red dot) by traversing the cluttered area (red cylinders) as fast as possible.

The BARN dataset is configured for a Clearpath’s Jackal robot, which has dimensions 508×430 mm. This robot is simulated using Gazebo [50] and the robot operating system (ROS) version, Noetic [51]. The robot is equipped with a 270${}^{\circ }$ field of view planar LIDAR. The proposed methodology was implemented in C++ and made publicly available at https://bitbucket.org/wvufarolab/sslat_planning/ (accessed on 21 June 2022). All tests of this section were executed on a computer with an Intel^®^ Core™ i9 at 3.6 GHz, sixteen cores, and 32 GB of main memory, equipped with an NVIDIA^®^ GeForce^®^ RTX 2080 with compute capability 7.5, GPU clock of 1.5 GHz, and global memory size of 8 GB. The maximum grid dimensions are [2,147,483,647, 65,535, 65,535], while the maximum block dimensions are [1024, 1024, 64] with a maximum number of threads per block of 1024. The dataset consists of 300 randomly generated environments of increasing difficulty according to 5 proposed metrics: distance to closest obstacle, average visibility, dispersion, characteristic dimension, and tortuosity. An example of a simulation environment provided by the dataset is shown in Figure 9.

**Figure 10:**
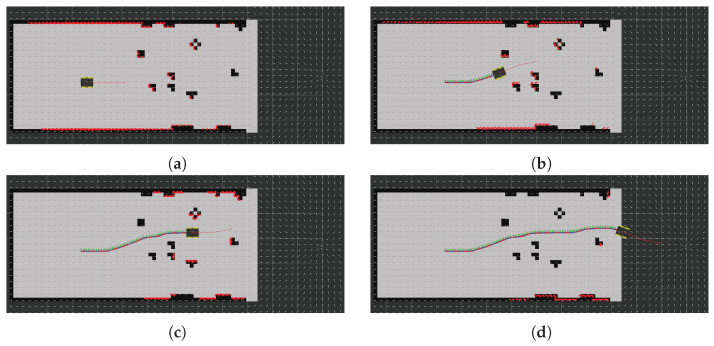
From (**a**–**d**), four snapshots of the solution provided by SSLAT in environment #6 of the BARN dataset (see Figure 9). The planned path is shown in red. The executed path is shown by a sequence of reference coordinate frames. A close inspection shows that the path of the robot follows the vector field whenever there are no interfering obstacles. Notice that the occupancy grid of the environment is shown for just to illustrate the scenario, and it is not used by SSLAT.

To test the proposed methodology, from the start position ${\left(x,y\right)}_{W}=\left(0,0\right)$, the robot was directed to the goal position ${\left(x,y\right)}_{W}=\left(0,10\right)$ using a constant vector field that simply sends the robot straight in the y direction, ${\left(u,v\right)}_{W}=\left(0,1\right)$, while crossing the field of obstacles, and another vector field pointing toward the goal afterwards. The lattice was configured using $\left(K,N_{T},N_{B},N_{L},r_{0}\right)=\left(2,16,3,3,0.4\right)$ and a collision radius of 0.35 m. The robot was controlled using an in-house developed path-following node that generates forward linear velocity ($v_{x}$) and yaw angular rate ($\omega_{z}$). Figure 10 shows four snapshots of a simulation that shows the robot successfully traversing the obstacle field. This figure also shows the vector field, the sensor measurements, the instantaneous local plan provided by SSLAT, and the path executed.

We simulated five runs of each of the first 100 environments of the BARN dataset using two different robot speeds: 0.50 and 1.15 m s^−1^. For these speeds, the optimal time the robot would take to go from the initial position (0,0) to the goal area (a 1 m radius centered in (0,10)) are 18.0 and 7.8 s, respectively, if the robot moves in a straight line. To compare our results, we also tested two baseline algorithms provided by the authors of the dataset, dynamic window approach (DWA) [26] and elastic bands (EBand) [21], using ROS *move_base* as a navigation framework. In this framework, a higher-level planner (A* in our experiments) is responsible for planning a path on a map created online using simultaneous localization and mapping (SLAM). DWA and EBand thus act as lower-level approaches that follow the path while avoiding previously undetected obstacles. The experiments were performed without tuning the parameters of each method for each environment.

#### 5.1.2. Results

**Figure 11:**
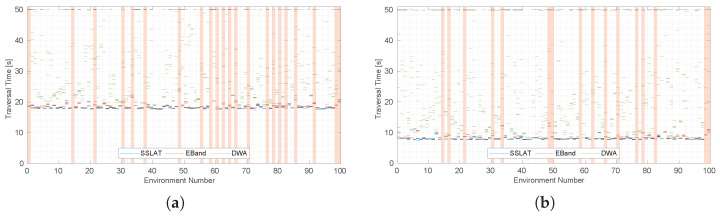
Comparison of three motion planning strategies using the first 100 environments of the BARN dataset [49]: SSLAT (blue), EBand (red) and DWA (green). In (**a**) the robot speed is limited to 0.50 m s^−1^ while in (**b**) it is limited to 1.15 m s^−1^. The results for the 5 runs of each environment are shown for each method. In case of collision or timeout, the traversal time was set to 50 s. The highlighted environments are the ones for which SSLAT was not able to find a solution.

Figure 11 shows the traversal times for each of the methods using 0.50 (a) and 1.15 m s^−1^ (b). In this figure, runs for which collisions happened were given a total traverse time of 50 s, as proposed by the authors of the dataset. Tests would also timeout at 50 s if the planners were taking too long to find a solution. Table 1 shows numerical details about the experiment.

We observe from Figure 11 and Table 1 that DWA, EBand and SSLAT are consistent in finding solutions for the environments tested. DWA presents a great time dispersion, since it frequently slows down the robot to find solutions. EBand and SSLAT can obtain results close to the optimum (18.0 and 7.8 s) when they find a solution. However, notice in Table 1 that none of the methods had 100% success in traversing all the environments. SSLAT is not able to solve every environment mostly because it does not have enough information to deal with dead ends (the vector field, which acts as a higher-level planner, does not use a map and ignores all the obstacles). The other two methods rely on a global path planner, so it was expected that they would perform better in the dataset.

In addition to the time of task completion, we also recorded the planning time for SSLAT and EBand. SSLAT has a maximum planning time of 3.28ms while EBand has a maximum planning time of 4.19ms.

### 5.2. Simulations with a Ground Robot in a Dynamic Environment

This subsection shows the performance of SSLAT in a modified version of the BARN environment, which was adapted to contain moving obstacles.

#### 5.2.1. Experimental Setup

**Figure 12:**
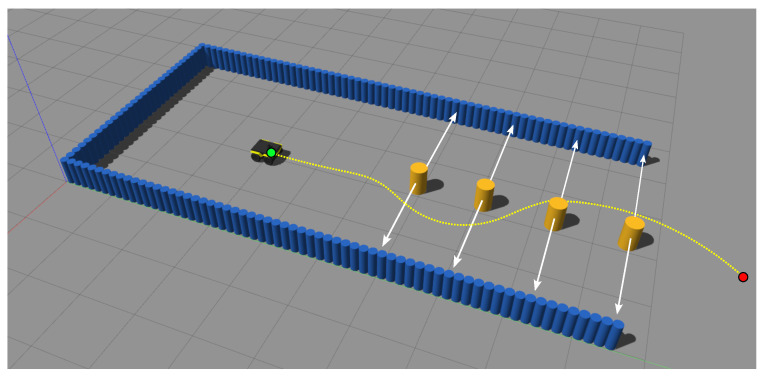
Environment used to test obstacle avoidance with dynamic obstacles. While the robot moves toward the goal, the four yellow obstacles move back and forth with random velocities.

In the environment used in this section, four moving cylinders were placed between the start and goal positions, as shown in Figure 12. The cylinders move side-to-side, each with a constant speed sampled uniformly from a speed interval at the beginning of each trial. Once the obstacles reach the wall, they switch directions, going back and forth. Since the robot is non-holonomic and cannot move sideways, the obstacles stop moving once the robot’s sensor passes by them, thus preventing unavoidable lateral collisions caused by the obstacles. Similar to the previous subsection, three methods were tested: SSLAT, EBand, and DWA. Each method was tested for two robot maximum speeds (0.50 and 1.15 m s^−1^) and three intervals for the speed of the obstacles: [0.00, 0.75] m s^−1^, [0.25, 1.00] m s^−1^, and [0.50, 1.25] m s^−1^. For comparison, a person tends to walk at around 1.3 m s^−1^ [52]. The same vector field and lattice configurations defined for the static environment in the previous subsection were used in the experiments of this subsection.

#### 5.2.2. Results

The success rate of the three planners for all situations tested is shown in Table 2. In this table, successful situations are the ones for which the robot reached the goal without collision in less than 100 s. We run each situation 50 times for each method. Table 2 also shows the average traversal time for all methods and its standard deviation. SSLAT was able to handle moving obstacles better than EBand but was overcome by DWA, as seen by the success rate. Notice that this happens on the expense of a increased traversal time by DWA, which reduces the robot speed to avoid the obstacles, while SSLAT and EBand try to keep the robot at maximum speed. Further, it is noticeable that all three methods are susceptible to failure due to the slow dynamics of the robot, i.e., even if the plan is updated fast, the robot does not necessarily follow that change.

### 5.3. Validation and Evaluation of the Parallel Implementation

This subsection presents the results of a ground robot navigating in a forest-like environment using the proposed methodology. Our goal in this section is to evaluate the parallelization of the tree pruning step of our algorithm by analyzing the effects of changing the density of obstacles in the environment and the number of triangles on the lattice.

#### 5.3.1. Experimental Setup

In this subsection, SSLAT was tested on a laptop computer with an Intel^®^ Core™ i7 at 2.4 GHz, eight cores, and with 16 GB of main memory and an NVIDIA^®^ GeForce^®^ GT 740 M with compute capability 3.5, two CUDA cores, GPU clock of 1 GHz, and global memory size of 2 GB. The maximum grid dimensions are [2,147,483,647, 65,535, 65,535], while the maximum block dimensions are [1024, 1024, 64] with a maximum number of threads per block of 1024. The total amount of shared memory per block is 48 kB and per-thread is 16 kB. Notice that, different from the powerful desktop computer used in the previous section, the computer used in this section could be carried by a ground robot in real life.

**Figure 13:**
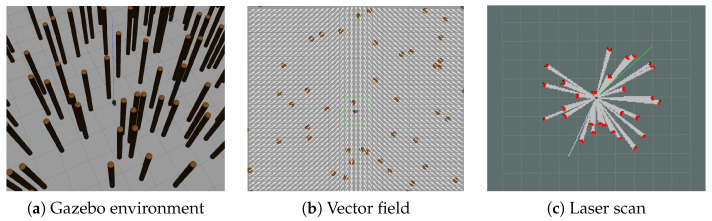
Experimental setup used to compare the performance of the parallel and serial versions of the SSLAT motion planner. In (**a**), the environment was constructed using cylindrical objects in Gazebo, to simulate a forest. The global task is encoded by the vector field in (**b**), which follows a straight line in the vertical direction. In (**c**) it is possible to see, in a specific robot position, the valid LIDAR scans (i.e., within the sensor range) used to prune the search tree.

Our experiments were executed using Gazebo simulations and ROS Melodic. In this test, a simulated ground robot was equipped with a planar LIDAR. The LIDAR was set to have 3.5 m of range and 360 laser beams (1 deg resolution). Figure 13 shows a typical environment, the vector field used to make the robot move across the forest, and an example laser scan measurement obtained while the robot is moving amidst the obstacles.

The obstacles consist of cylinders randomly placed on the map to simulate a Poisson forest of different densities, as proposed by [53]. In our experiments, we varied the density of the forest from 0.1 to 3.2. To vary the number of triangles in our method, the number of trunks, $N_{T}$, was changed from 8 to 64 in powers of 2, and the number of layers, $N_{L}$, varied between 4 and 5. All the other parameters were kept constant as $\left(K,N_{B},r_{0}\right)=\left(2,3,0.4\right)$. The collision detection radius was set to 0.20 m.

#### 5.3.2. Results

**Figure 14:**
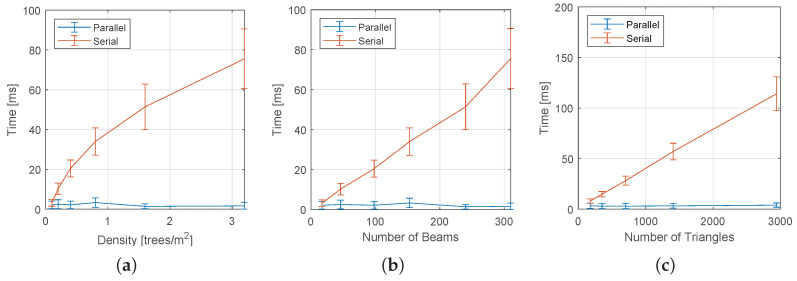
Effect of the lattice parameters and environment density on the tree pruning time for both the serial and parallel implementation of the method. (**a**) Effect of the density of the forest; (**b**) effect of the number of laser beams; (**c**) effect of the number of triangles.

**Figure 15:**
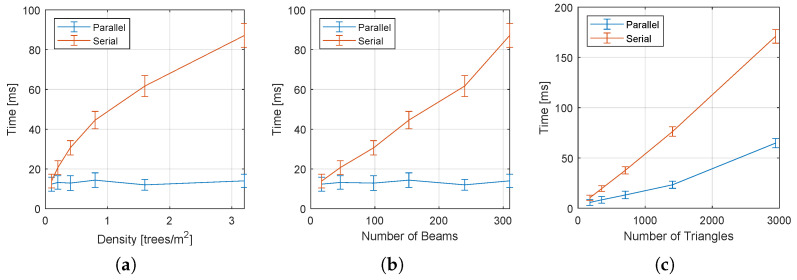
Effect of the lattice parameters and environment density on total planning time for both the serial and parallel implementation of the method. (**a**) Effect of the density of the forest; (**b**) effect of the number of laser beams; (**c**) effect of the number of triangles.

Figure 14 shows the effect of the forest density and the number of triangles in the lattice on the collision-check time used for pruning the search tree. In Figure 15, we have similar graphs for the complete algorithm.

As the density increases, more laser beams are considered valid, causing the collision checking time in the serial implementation to grow monotonically. Notice in Figure 14b that the time grows almost linearly with the number of valid LIDAR beams. On the other hand, the parallel version maintains an almost constant time, independently of the density, number of beans, and number of triangles. As a result, for large obstacle densities, the speed up of the parallel implementation is greater than 25.

Notice that for the complete algorithm (Figure 15), the effect of the forest density leads to conclusions similar to the observed for the collision checking time (Figure 14). However, there is an important difference in the graph that measures the effect of the number of triangles, where the total time also increases in the parallel version. This is because cost assignment, which was not parallelized in our implementation, grows linearly with the number of edges. This suggests that future implementations should also consider the parallel implementation of this step, which can be easily accomplished using the algorithm presented in [54].

### 5.4. Experiments with a Real-World UAV

This subsection presents a real-world experiment that shows SSLAT flying a drone under the canopy of a sparse forest.

#### 5.4.1. Experimental Setup

The robot used in this section is a DJI Matrice 100 quadrotor. This drone has a built-in navigation solution based on the global positioning system (GPS) and inertial measurement unit (IMU) that provides the vehicle localization at 50 Hz. The drone is actuated by its linear velocities and angular rate. For our experiment, the drone was equipped with a UDOO X86 (CPU Intel Pentium N3710 2.56 GHz, 8 G RAM) single board computer running Linux, which communicates with the drone using a ROS driver provided by the maker. Since the drone’s onboard computer is not compatible with CUDA, our experiments ran the sequential, non-parallel version of SSLAT. Besides the computer, our drone was equipped with an Intel^®^ RealSense™ D435i camera, which provides depth images at 30 Hz. Since our algorithm expects 2D LIDAR data, a ROS node (*depthimage_to_laserscan*) was used to transform the camera output into the required input for our software.

**Figure 16:**
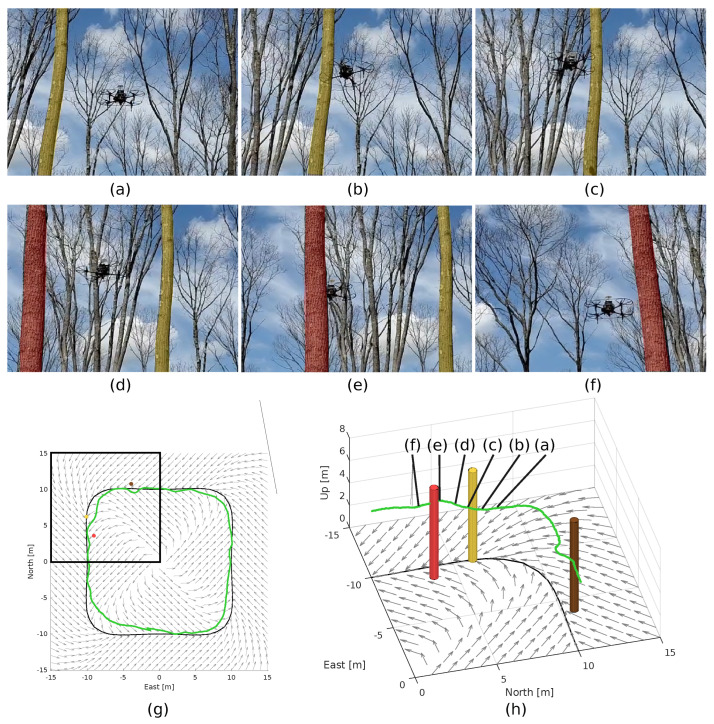
Results of an experiment performed with a drone in a forest. (**a**–**f**) shows snapshots of the part of the experiment highlighted in the black box of (**g**). In (**g**,**h**), the vector field is shown by the gray arrows, the target curve is represented by the black curve, and trees are represented by cylinders. The cylinders were painted in yellow and red to facilitate the correspondence with the actual trees in the snapshots (**a**–**f**), which were also painted with the same colors. The executed trajectory, obtained from the localization system of the drone, is shown in green. In this trajectory, the letters (**a**–**f**) correspond to the snapshots shown.

In our experiment, we designed a vector field to circulate a closed curve shaped as a square with rounded corners and parameterized by the potential field $\phi \left(x,y\right)={\left(x/10\right)}^{4}+0.5{\left(x/10\right)}^{2}{\left(y/10\right)}^{2}+{\left(y/10\right)}^{4}-1$ (see Figure 16g). The computation of such an artificial vector field was shown in [45]. The lattice was configured with $\left(K,N_{T},N_{B},N_{L},r_{0}\right)=\left(2,16,3,3,0.5\right)$ and a collision radius of 0.60 m.

Instead of using a path following controller, in this experiment, the drone was controlled using linear horizontal velocities ($v_{x}$ and $v_{y}$) and the yaw angular rate ($\omega_{z}$) obtained from the first waypoint of the path planned by SSLAT, represented by $\left(x_{sp},y_{sp}\right)$ in the robot reference frame. The height was kept constant. The velocities commanded to the drone were computed as

(4) $$v_{x}=V\frac{x_{sp}}{\sqrt{x_{sp}^{2}+y_{sp}^{2}}},\;v_{y}=V\frac{y_{sp}}{\sqrt{x_{sp}^{2}+y_{sp}^{2}}},\;v_{z}=0,\;\omega_{z}=K_{\psi }\psi_{sp},$$

where *V* is the constant linear speed, set to be 1.5 m s^−1^. The angular speed was set to be proportional to the error in yaw angle given by $\psi_{sp}=\arctan2\left(y_{sp},x_{sp}\right)$. This maintains the robot pointing to the direction of the planned path, which is important due to the limited field of view of the sensor used (87${}^{\circ }$).

#### 5.4.2. Results

Figure 16 shows six snapshots of our experiment along with the trajectory of the drone obtained by its localization system. Since the forest was sparse and had almost no leaves, the GPS information was available during the entire flight. The results of Figure 16 show that the drone successfully tracked the target curve guided by the vector field, avoiding the trees when this was necessary. This suggests that the proposed approach can be used to provide safe motion at fairly high speeds (1.5 m s^−1^), even in challenging outdoor environments, such as a forest.

## 6. Discussion

Our experimental results show that SSLAT has the potential to be used to guide fast robots to perform a variety of tasks. We benchmarked SSLAT against two well-known and optimized strategies available in ROS. Our numerical results showed that SSLAT, using a very simple vector field as a global planner, is slightly inferior to EBand when dealing with static environments, but much faster than DWA in the same situations. At this point, notice that the success rate of our planner could be improved if we opted to create a vector field using knowledge about the environment, which would be equivalent to the high-level planner used by EBand and DWA. On the other hand, in the presence of moving obstacles, the success rate of SSLAT is greater than the one observed for EBand and is less than the one observed for DWA, which tends to reduce the robot speed to avoid obstacles.

Our results also show that a robot running SSLAT (and also EBand and DWA) may not be able to avoid every possible collision, indicating that it would need to be complemented by other strategies. In the simulations presented in this paper, the robot is not equipped, for example, with an emergency stop behavior that would stop the robot in the eminence of a collision. Conversely, SSLAT tries to keep the robot velocity at its maximum to reduce the time it traverses the environment. This behavior is risky and would not be acceptable in most real-world applications. Additionally, we notice that, although SSLAT computes a free-of-collision path at each iteration, the controller used not always can follow the path. This suggests that better path-following controllers need to be used.

The main goal of the experiments presented in this paper was to show the potential of SSLAT for fast path computation. Although the maximum planning times for both SSLAT and the baseline, EBand, are very small, indicating that both strategies can be used in hard real time, notice that EBand was run on an Intel i9 CPU in our experiments, what may not be available for most robots. On the other hand, since the most expensive routine of SSLAT runs on the GPU, we expect that its planning time will not increase too much when it runs on embedded GPUs, such as, for example, the NVIDIA^®^ Jetson^®^.

In our last experiment, we show SSLAT controlling a drone in a challenging forest environment, in the presence of environmental disturbances (i.e., wind, shade, and direct sun light). Although we have not considered uncertainties, disturbances and failures explicitly in the design of our method, SSLAT is inherently robust to such input issues once it is a closed-loop approach at all its levels, and most importantly, it runs fast. First, notice that the vector field is a closed loop since for each position in the space, we have a velocity vector. Moreover, most vector fields are stable in the sense of Lyapunov [10,13]. In the avoidance level, any disturbances, such as new obstacles, moving obstacles, and changes in the position of the vehicle due to wind are rapidly detected in the lattice, and a new path is created. This process happens in a few milliseconds, which is enough to reject the most common disturbances. In addition to external disturbances, SSLAT presents some robustness to sensor uncertainties. For each sensor beam, it tests the collision between a circle, initially defined by the radius of the robot, and the triangles of the lattice. By increasing the radius of the circle, we can account for the uncertainty of the sensor, consequently being more conservative and moving farther from the obstacles. We can also observe some robustness to sensor failures that could lead to missing obstacle detection. Because we may have several LIDAR beams associated with each triangle, a failure in a single beam would not be a problem, especially for obstacles close to the robot, since the edges of the graph that would lead to collisions could be pruned by another beam. Naturally, the method would fail if an obstacle is not detected by any beam, what may happen when the obstacle is small and is far from the robot. This, however, is a limitation of the sensor, and not of the method.

## 7. Conclusions and Future Work

Motion planning in real time is an important requirement for the navigation of fast robots in cluttered environments. This paper presented the SSLAT motion planner, which is an embarrassingly parallel strategy that computes local paths within the sensor space of a robot. With our current CUDA hardware, we were able to compute paths using SSLAT as fast as 2 ms (500 Hz). This is just a fraction of the sampling time of a typical range sensor, which operates in frequencies that range from 10 to 100 Hz. So, SSLAT can compute paths in hard real time, what makes it as a good candidate strategy to be used in static and dynamic environments, especially in applications where the main direction of movement can be defined by a vector field. Although this seems to be limiting, there are several common tasks, such as road or corridor following [18,28], forest traversing [17], or perimeter surveillance [55] that lie in this category.

This paper showed the application of SSLAT for ground and aerial vehicles operating in cluttered environments. By benchmarking SSLAT against baseline approaches using the BARN dataset, we showed that it is approximately 2.3 times faster than DWA, but, overall, it leads to approximately 21% more collisions than EBand while moving at similar speeds. This occurs because EBand uses a global optimum planner, while the vector field used by SSLAT was set to be a simple one that only moves the robot forward. By comparing SSLAT with the same baseline approaches in a dynamic environment, we show that its success rate is greater than that of EBand (58% versus 46% for high robot and obstacle speeds), but it is less than that of DWA (58% versus 80%). However, we noticed that DWA is more conservative, leading to completion times approximately 19% higher than the ones archived with SSLAT. Overall, we then conclude that SSLAT would be a competitive choice if both static and moving obstacles need to be avoided at fast speeds.

Although our results showed the efficacy of the methodology for obstacle avoidance, we see some potential for improvement. First, observe that the paths created by SSLAT are not smooth, which can be demanding for the robot controller. To solve this, a post-processing step could be applied to the final path or, more interesting, a smooth lattice [33] could be developed and applied. Second, we noticed a few situations in our experiments, where the robot became undecided in front of an obstacle. Because the path is computed much faster than the robot can follow it, in very symmetric situations, SSLAT computes consecutive paths that have different homotopies (e.g., one path avoids the obstacle by the right and the other by the left). We usually solved that by slowing down the path computation to allow the robot to follow the computed path. A better strategy would be to slightly modify our cost functional to prioritize paths that are homotopic to the previous one. Finally, our method finds paths in 2D. Although we used SSLAT to control drones, future work could include expanding it to 3D. Giving the sensor space an extra dimension will increase the number of triangles (or perhaps tetrahedrons in this case) to be tested. The parallelism of the collision check should be as simple as in the 2D case, but hardware limitations may appear and, consequently, multidimensional CUDA grids and blocks could be beneficial.

## Figures and Tables

**Table 1:** Comparison of three motion planning strategies using the first 100 environments of the BARN dataset [49]. Each method was run five (5) times for each environment. The mean and standard deviation values only consider successful (no collisions/no timeout) trials.

Method	Robot Speed [m s^−1^]	Success Rate [%]	Environments with Success in All Five Trials [%]	Environments with Failure in All Five Trials [%]	Traversal Time [s]	
					**Mean**	**Std. Dev.**
SSLAT	1.15	71.8	10.6	4.4	8.540	2.000
	0.50	69.4	12.0	28.2	18.722	2.495
EBand	1.15	93.4	16.4	0.4	8.540	0.931
	0.50	97.2	18.4	0.0	18.604	0.945
DWA	1.15	93.6	15.6	0.0	19.787	8.478
	0.50	97.4	18.2	0.0	27.604	7.493

**Table 2:** Performance comparison of three motion planning strategies in the presence of moving obstacles, as shown in Figure 12. The speed of the obstacles is randomly sampled from the following intervals: Low [0.00, 0.75] m s^−1^; Mid [0.25, 1.00] m s^−1^; Fast [0.50, 1.25] m s^−1^.

Method	Robot Speed [m s^−1^]	Obstacle Speed [m s^−1^]	Success Rate [%]	Traversal Time [s]	
				Mean	Std. Dev.
SSLAT	0.50	Slow	72	19.428	1.146
		Mid	38	19.661	0.899
		Fast	44	19.951	0.797
	1.15	Slow	72	8.960	1.140
		Mid	60	9.090	1.060
		Fast	58	8.970	0.890
EBand	0.50	Slow	58	19.041	0.918
		Mid	32	19.860	1.128
		Fast	24	19.931	0.910
	1.15	Slow	58	8.800	0.710
		Mid	66	8.910	0.640
		Fast	46	9.230	0.870
DWA	0.50	Slow	82	21.625	3.785
		Mid	62	20.833	2.230
		Fast	58	20.845	1.724
	1.15	Slow	92	11.730	4.330
		Mid	82	10.310	1.870
		Fast	80	10.660	1.450

## Data Availability

The data presented in this study are available on request from the corresponding author. The software developed is openly available at https://bitbucket.org/wvufarolab/sslat_planning/, accessed on 21 June 2022.

## Abbreviations

The following abbreviations are used in this manuscript:

SSLAT	Sensor Space Lattice
RRT	Rapidly Exploring Random Tree
PRM	Probabilistic Roadmap
GMT	Group Marching Tree
LIDAR	Light Detection and Ranging
CTG	Cost-to-go
GPU	Graphics Processing Unit
SLAM	Simultaneous Localization and Mapping
CPU	Central Processing Unit
CUDA	Compute Unified Device Architecture
ROS	Robot Operating System
BARN	Benchmark for Autonomous Robot Navigation
APF	Artificial Potential Function	
VFF	Virtual Force Field
EBand	Elastic Bands
VFH	Vector Field Histogram
GPS	Global Positioning System
DWA	Dynamic Window Approach
IMU	Inertial Measurement Unit
CDC	Collision Detection Circuit
2D/3D	Two/Three-Dimensional
